# Metformin Prevents Fatty Liver and Improves Balance of White/Brown Adipose in an Obesity Mouse Model by Inducing FGF21

**DOI:** 10.1155/2016/5813030

**Published:** 2016-01-19

**Authors:** Eun Kyung Kim, Seung Hoon Lee, Joo Yeon Jhun, Jae Kyeong Byun, Jeong Hee Jeong, Seon-Young Lee, Jae Kyung Kim, Jong Young Choi, Mi-La Cho

**Affiliations:** ^1^The Rheumatism Research Center, Catholic Research Institute of Medical Science, The Catholic University of Korea, Seoul, Republic of Korea; ^2^Impact Biotech, Korea 505 Banpo-Dong, Seocho-Ku, Seoul 137-040, Republic of Korea; ^3^Division of Hepatology, Department of Internal Medicine, College of Medicine, Seoul St. Mary's Hospital, The Catholic University of Korea, 505 Banpo-Dong, Seocho-Ku, Seoul 137-040, Republic of Korea; ^4^Division of Rheumatology, Department of Internal Medicine, The Catholic University of Korea, Seoul 137-040, Republic of Korea

## Abstract

Obesity and its associated metabolic disorders are related to the onset of fatty liver and the balance of white adipose tissue (WAT) and brown adipose tissue (BAT). We hypothesized that metformin, an effective pharmacological treatment for type 2 diabetes, would inhibit white adipogenesis, fatty liver, and metabolic dysfunction. Metformin was treated daily for 14 weeks in a high-fat dieting C57BL/6J mice. Serum biomarkers were analyzed and protein level was assessed using confocal staining or flow cytometry. The development of lipid drops in the liver cells and white adipocyte was measured using hematoxylin and eosin or Oil Red O stains. Gene expressions were analyzed with quantitative real-time PCR. Metformin treatment decreased the body weight and improved the metabolic profile of obese mice. In obese mice, metformin also induced the expression of BAT-related markers and increased fibroblast growth factor (FGF) 21 expression in the liver and in white adipocyte. Metformin suppressed white adipocyte differentiation via induction of FGF21. Metformin improves Treg/Th17 balance in CD4+ T cells in mice with high-fat diet-induced obesity. Metformin also improves glucose metabolism and metabolic disorder. Interleukin-17 deficiency also decreases inflammation in mice. Therefore, metformin may be therapeutically useful for the treatment of obesity and metabolic dysfunction.

## 1. Introduction

Obesity is a medical condition in which surplus body fat increases to the point where it negatively affects health. Obesity reduces life expectancy and leads to metabolic problems; for example, obesity results in high levels of triglyceride (TG) and low levels of high density lipoprotein- (HDL-) cholesterol in plasma [1]. On the other hand, dense low-density lipoprotein (LDL) particles are enhanced in obese patients [2]. It has been reported that obesity is associated with liver abnormalities such as fatty liver [3]. Obesity is also related to inflammation. Several publications have demonstrated that obesity increases proinflammatory cytokine expression and reduces anti-inflammatory cytokine production [4, 5]. It is well documented that obesity is involved in inflammatory and autoimmune diseases associated to interleukin- (IL-) 17-producing T (Th17) cells, and this increases the probability of several diseases including type 2 diabetes and rheumatoid arthritis [6–8].

Metformin is a biguanide antidiabetic drug that has successfully been used to treat patients with type 2 diabetes. Recently, it has been suggested that metformin could be used to treat other diseases. Metformin inhibited the inflammatory response that regulates Th17 cells and regulatory T (Treg) cells in a rheumatoid arthritis mouse model [9]. Metformin has also been demonstrated to have an anti-inflammatory activity in an inflammation-associated tumor mouse model [10].

Fibroblast growth factor (FGF) 21 is a metabolic hormone that is generated mainly in the liver. It has been shown that FGF21 prompts glucose uptake in adipocytes and improves metabolic markers such as blood glucose, insulin, and triglycerides (TG) [11]. FGF21 positively affects the metabolic profile of mice with diet-induced obesity [12]. Moreover, FGF21 has emerged as a significant metabolic regulator and contributes to the control of lipolysis in white adipose tissue (WAT) [13, 14]. Previous research has suggested that FGF21 is a potential therapeutic effect for the treatment of human diabetes and obesity [15].

We hypothesized that metformin would improve the metabolic profile of mice with high-fat diet-induced obesity. The present study attempts to determine whether metformin functions as a treatment for obesity by inducing the expression of FGF21. To test this hypothesis, we evaluated the therapeutic role of metformin in obesity and investigated its effect on metabolic profile and Th17-mediated inflammation in a mouse model of obesity.

## 2. Materials and Methods

### 2.1. Animals

C57BL/6 mice (Male, Orient Bio, Republic of Korea), aged 4 weeks old, were maintained under specific pathogen-free conditions and fed a diet containing 60 kcal fat-derived calories or standard laboratory mouse chow (Ralston Purina, St. Louis, MO, USA) and water ad libitum (*n* = 10). All experimental procedures were examined and approved by the Animal Research Ethics Committee of the Catholic University of Korea (permit number: CUMC-2015-0009-01), which conforms to all National Institutes of Health of the USA guidelines. All surgeries were performed under isoflurane anesthesia, and all efforts were made to minimize suffering. The experimental protocol was approved, and all animals were treated and sacrificed in accordance with the guidelines of the Catholic University of Korea on Use and Care of Animals.

### 2.2. Metformin Treatment

Metformin, dissolved in saline, was orally administered daily for 14 weeks. Metformin was administered at a dose of 10 mg/kg or 50 mg/kg. The control mice were administered with saline.

### 2.3. Cell Culture

HepG2 cells and 3T3L1 cells were purchased from the Korean cell line bank and maintained in minimum essential medium (MEM) or Dulbecco's modified Eagle's medium (DMEM) containing 10% fetal bovine serum (FBS) in an incubator with 5% CO_2_ at 37°C. The cells (1 × 10^4^ cells/well) were cultured in 24-well plates in serum-free MEM media or differentiation medium plus metformin (5 mM) or anti-FGF21 for 24 h.

Murine 3T3-L1 preadipocytes were cultured in preadipocyte medium (24-well plates, 1 × 10^4^ cells/well) and allowed to reach 100% confluence. Once the cells are confluent, incubate for an additional 48 hours before initiating differentiation. Two days after the cells have been confluent replace with an appropriate volume 3T3-L1 differentiation medium. Incubate for 3 days, and replace with 3T3-L1 adipocyte maintenance medium.

### 2.4. Confocal Microscopy

For immunostaining, 7 *μ*m tissue sections of spleen were stained using fluorescein isothiocyanate- (FITC-) conjugated anti-CD4, phycoerythrin- (PE-) conjugated anti-IL-17, allophycocyanin- (APC-) conjugated anti-CD25, PE-conjugated anti-Foxp3 (all from eBiosciences, San Diego, CA, USA), anti-FGF21 (Bioss), and anti-CoxIV (cell signaling). The stained sections were analyzed using a Zeiss microscope (LSM 510 Meta; Carl Zeiss, Oberkochen, Germany) at 400x magnification.

### 2.5. Biochemical Analyses

Blood samples were collected in serum tubes from all treated and control mice at 14 weeks and stored at −70°C until use. The levels of total serum cholesterol were measured using commercial kits (Wako Co., Osaka, Japan). AST, ALT, glucose, and LDL-cholesterol levels were measured using commercial kits from Asan Pharmaceutical Co. (Hwangseong-gi, Gyeonggi-do, Republic of Korea). The levels of serum were measured using a Hitachi 7600 analyzer (Roche).

### 2.6. Glucose Tolerance and Insulin Tolerance Tests

For the insulin tolerance test, nonfasted mice were injected intraperitoneally injection of insulin (1 U/kg body weight). For glucose tolerance test, mice were fasted overnight and then loaded intraperitoneally with injection of glucose (1 g/kg body weight).

### 2.7. Intracellular Staining and Flow Cytometry

We investigated changes in the population of Foxp3-positive Treg cells and Th17 cells after metformin treatment. To analyze intracellular cytokines, cells were stimulated with 25 ng/mL PMA and 250 ng/mL ionomycin (all from Sigma-Aldrich, St. Louis, MO, USA) and Golgi Stop (BD Biosciences, San Diego, CA, USA) in a 24-well plate and incubated for 4 h. Splenocytes were stained with Percp-conjugated anti-CD4 antibody, followed by fixation and permeabilization using the Cytofix/Cytoperm Plus Kit (BD Biosciences) according to the manufacturer's instructions and then stained with FITC-conjugated anti-IL-17 (all from eBiosciences). For analysis of Treg cells, splenocytes were surface labeled with CD4 and CD25, followed by fixation, permeabilization, and intracellular staining with Foxp3 as per the manufacturer's protocol. All samples were examined using a FACSCalibur (BD Pharmingen), and the data was analyzed using FlowJo software (Tree Star, Ashland, OR, USA).

### 2.8. Analysis of Gene Expression by Quantitative Real-Time Reverse Transcriptase PCR (qRT-PCR)

mRNA was extracted using TRIzol (Molecular Research Center). cDNA was synthesized using the Superscript Reverse Transcription system (Takara, Shiga, Japan). qRT-PCR was performed with LightCycler FastStart DNA master SYBR green I (Takara), following the manufacturer's instructions. All mRNA expression levels were normalized to those of b-actin.

The primer sequences used were PPARr (forward: TCGCTGATGCACTGCCTATG, reverse: GAGAGGTCCACAGAGCTGAT); C/EBP-a (forward: CAAGAACAGCAACGAGTACCG, reverse: GTCACTGGTCAACTCCAGCAC); aP2 (forward: GATGCCTTTGTGGGAACCT, reverse: CTGTCGTCTGCGGTGATTT); Adipsin (forward: CATGCTCGGCCCTACATGG, reverse: CACAGAGTCGTCATCCGTCAC); Agt (forward: AACACCAGCATCCAGTTCAA, reverse: GGTTCAGTAGGCCATTCCTC); Pank3 (forward: TGCTGTAGTGTCCCATTTCTGCCT, reverse: AGCTGGAACAGCAACACCTAGGAA); Resistin (forward: AAGAACCTTTCATTTCCCCTCCT, reverse: GTCCAGCAATTTAAGCCAATGTT); Wdnm1L (forward: AGTGAGACCTCTGCAGCTTTTAGG, reverse: CAACTGTTTTCTTGGTCACAGAGC); Adiponectin (forward: GTCAGTGGATCTGACGACACCAA, reverse: ATGCCTGCCATCCAACCTG); Glut4 (forward: ACTCTTGCCACACAGGCTCT, reverse: AATGGAGACTGATGCGCTCT); Leptin (forward: CCTCATCAAGACCATTGTCACC, reverse: TCTCCAGGTCATTGGCTATCTG); LPL (forward: GGAAGAGATTTCTCAGACATCG, reverse: CTACAATGACATTGGAGTCAGG); UCP1 (forward: CTTTGCCTCACTCAGGATTGG, reverse: ACTGCCACACCTCCAGTCATT); Elvol3 (forward: CGGGTTAAAAATGGACCTGA, reverse: CCAACAACGATGAGCAACAG); Cidea (forward: GCCGTGTTAAGGAATCTGCTG, reverse: TGCTCTTCTGTATCGCCCAGT); Cox7a1 (forward: AGAAAACCGTGTGGCAGAGA, reverse: CAGCGTCATGGTCAGTCTGT); PGC1a (forward: GTCAACAGCAAAAGCCACAA, reverse: TCTGGGGTCAGAGGAAGAGA); PRDM16 (forward: GACATTCCAATCCCACCAGA, reverse: CACCTCTGTATCCGTCAGCA); IL-17 (forward: CCT CAAAGCTCAGCGTGTCC, reverse: GAGCTCACT TTTGCGCCAAG); Foxp3 (forward: GGCCCT TCT CCAGGACAG A, reverse: GCTGATCATGGCTGGGTTGT); *β*-actin (forward: GAAATCGTGCGTGACATCAAAG, reverse: TGTAGTTTCATGGATGCCACAG).

### 2.9. Statistical Analysis

The results are expressed as means ± SD (or means ± SEM). Statistical analysis was conducted using the Mann-Whitney *U* test using GraphPad Prism 5 software. *P* < 0.05 (2-tailed) was considered statistically significant.

## 3. Results 

### 3.1. Effect of Metformin Treatment on Body Weight and Adipose Tissue Weight in Mice with High-Fat Diet-Induced Obesity

**Figure 1:**
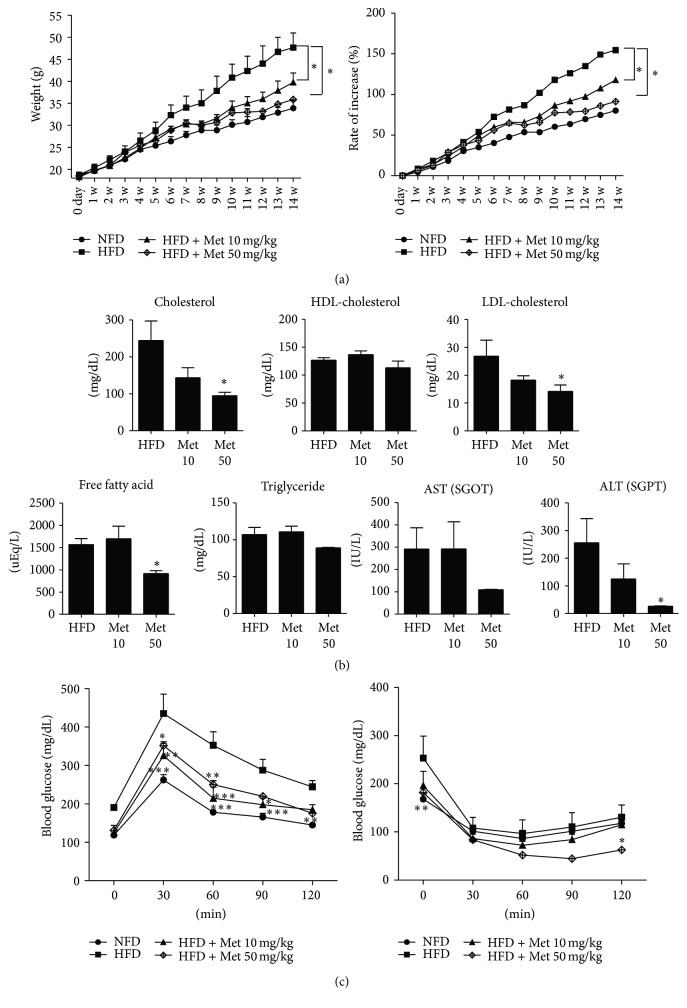
Effect of metformin in mice fed a high-fat diet. Obesity was induced in C57BL/6J mice and then 10 mg/kg or 50 mg/kg metformin (*n* = 10), or saline (*n* = 10) was administered orally on a daily basis to obese mice. (a) Body weight (mean ± SEM) was recorded in mice treated with metformin (10 mg/kg or 50 mg/kg) and compared to control mice. (b) Untreated obese mice or metformin-treated obese mice had different metabolic profiles. (c) Glucose tolerance test (GTT) and insulin tolerance test (ITT) in untreated obese mice and metformin-treated obese mice over 14 weeks.

To evaluate the antiobesity effect of metformin, mice were fed a high-fat diet (60 kcal) and were administered metformin (10 mg/kg/day and 50 mg/kg/day) or control (saline) orally. The metformin-treated group showed significantly reduced weight gain (Figure 1(a)). To investigate the effect of metformin, the metabolic profiles of mice were measured. The metformin-treated obese mice showed significantly lower total cholesterol, LDL-cholesterol, triglyceride, aspartate transaminase (AST), and alanine transaminase (ALT) levels in plasma than the untreated obese group (Figure 1(b)). The metformin-treated obese mice showed a decrease in blood glucose intolerance and insulin resistance levels (Figure 1(c)). These results suggest that metformin improves the health of high-fat diet-induced obese mice.

### 3.2. Effect of Metformin Treatment on Fatty Liver and White Fat/Brown Fat Balance in Mice with High-Fat Diet-Induced Obesity

**Figure 2:**
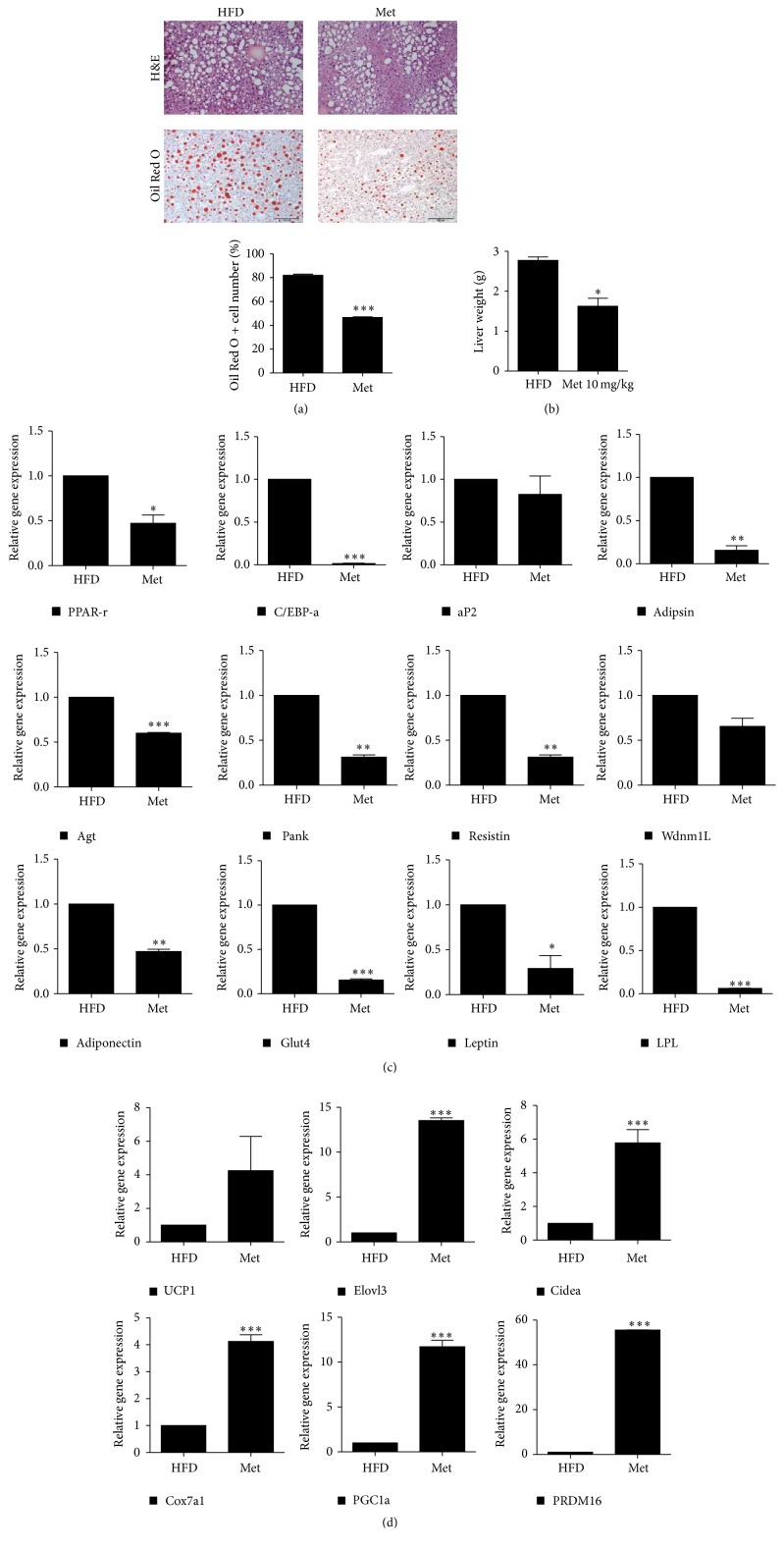
Metformin treatment ameliorates fatty liver. (a) Liver tissues were obtained from untreated obese mice and metformin-treated obese mice. Sections were stained with hematoxylin and eosin and Oil Red O stains. (b) Liver weight of untreated obese mice and metformin-treated obese mice. (c and d) Relative gene expression associated with (c) white adipose tissue (WAT) and (d) brown adipose tissue (BAT). Data represent means ± SEM. ^*∗*^ *P* < 0.05, ^*∗∗*^ *P* < 0.01, and ^*∗∗∗*^ *P* < 0.001.

To investigate the effect of metformin on high-fat diet-induced fatty liver in mice, we performed hematoxylin and eosin (H&E) and Oil Red O staining of the livers. Consequently, metformin-treated mice showed significantly decreased lipid content (Figure 2(a)). Moreover, the weight of the liver also decreased with metformin treatment (Figure 2(b)). Next, we examined the mRNA expression levels of the white adipose tissue and brown adipose tissue (BAT) in the livers. The expression levels of WAT-associated genes significantly reduced in the metformin-treated group; however, the expression levels of BAT-associated genes, such as UCP1, Elovl3, and Cidea, increased (Figures 2(c) and 2(d)).

### 3.3. Metformin Treatment Prevents the Development of Lipid Droplets in the Liver Tissue and Cells of Mice with High-Fat Diet-Induced Obesity via FGF21 Induction

**Figure 3:**
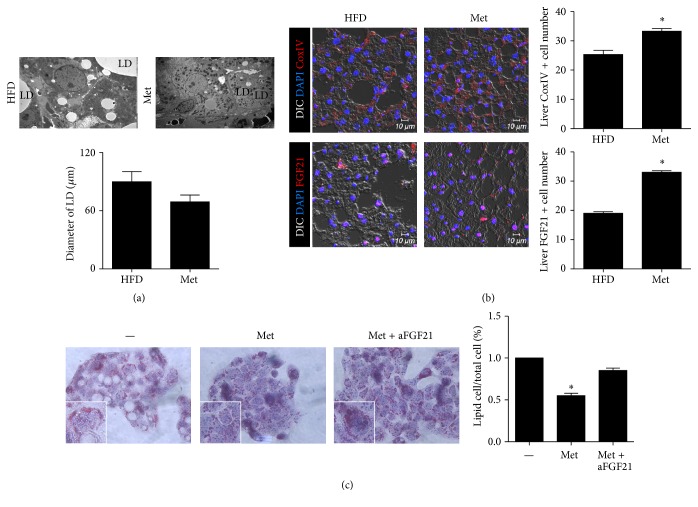
Metformin induced FGF21 production. (a) Electron microscopy of liver tissues from untreated obese mice and metformin-treated obese mice. Scale bars, 2 *μ*M. (b) CoxIV and FGF21 immunostaining of liver from untreated obese mice and metformin-treated obese mice. Scale bars, 10 *μ*M. (c) Representative Oil Red O staining in metformin (5 mM) or metformin plus anti-FGF21-treated HepG2 cells.

Previous studies have shown that the formation of lipid droplets is commonly associated with obesity. Therefore, we used transmission electron microscopy to show that the number of lipid droplets significantly increased in the untreated obese mice compared to the metformin-treated group (Figure 3(a)). To investigate the mechanism by which metformin regulates obesity, we analyzed the expression of CoxIV and FGF21 using confocal staining. Notably, FGF21 expression was upregulated in the livers of the metformin-treated group (Figure 3(b)). To confirm FGF21 function, we stained HepG2 cells with Oil Red O after HepG2 cells were treated with metformin, both in the presence and absence of anti-FGF21. It was found that lipid drop formation increased in the metformin plus anti-FGF21 group but not in the metformin group (Figure 3(c)). These data suggest that metformin suppressed obesity through the activation of FGF21.

### 3.4. Effect of Metformin Treatment on Peritoneal Visceral Fat Regulation in Mice with High-Fat Diet-Induced Obesity

**Figure 4:**
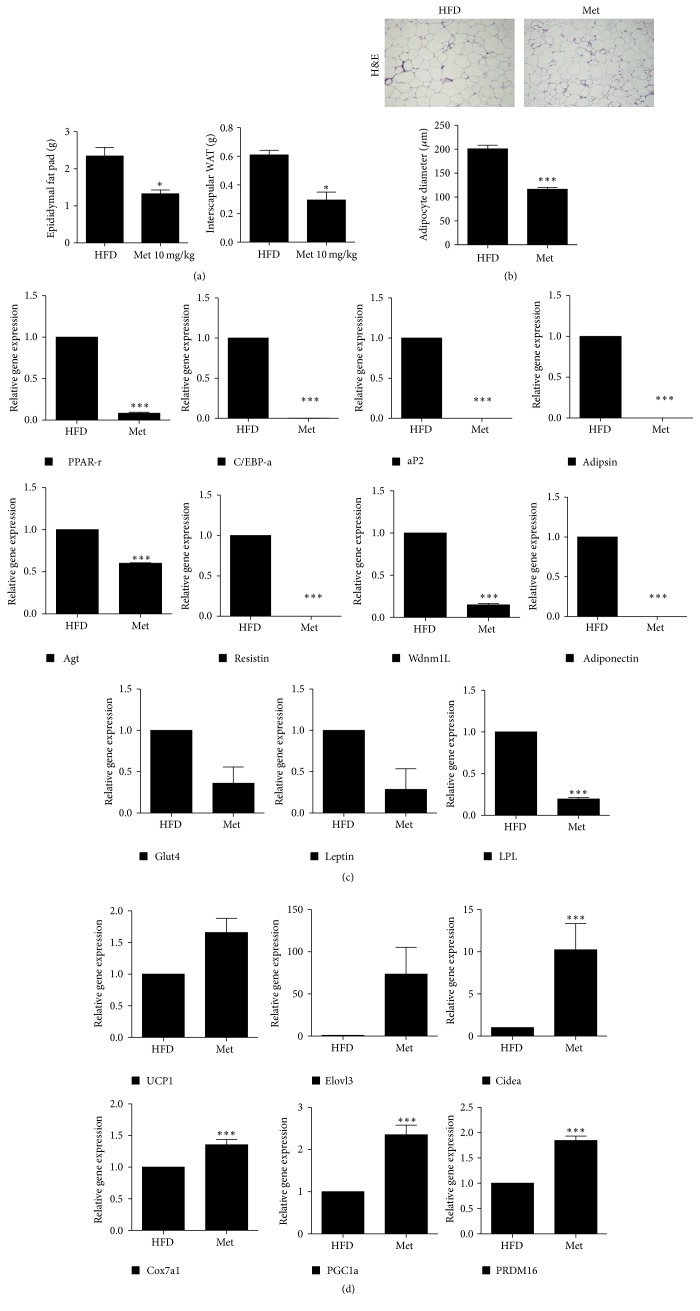
Metformin suppressed visceral fat and browning of white adipose tissue (WAT). (a) The epididymal fat and interscapular WAT weight in mice. (b) Visceral fat tissues hematoxylin and eosin staining. (c and d) Relative gene expression associated with (c) WAT and (d) brown adipose tissue (BAT) in visceral fat. Data represent means ± SEM. ^*∗*^ *P* < 0.05, ^*∗∗*^ *P* < 0.01, and ^*∗∗∗*^ *P* < 0.001.

We next measured the weight of epididymal fat and interscapular WAT and used H&E staining to further investigate the effect of metformin. It was found that the amount and size of fat was lower in the metformin-treated group than that in the untreated high-fat diet-fed mice (Figures 4(a) and 4(b)). We also examined the mRNA expression levels of WAT and BAT in the visceral fat. The expression levels of genes associated with WAT significantly reduced in the visceral fat of metformin-treated group; however, that of BAT-associated genes, such as UCP1, Elovl3, and Cidea, increased (Figures 4(c) and 4(d)).

### 3.5. Metformin Treatment Controls Reciprocal Positive Regulation between White Fat and Brown Fat via FGF21 Induction in 3T3L1 Cells

**Figure 5:**
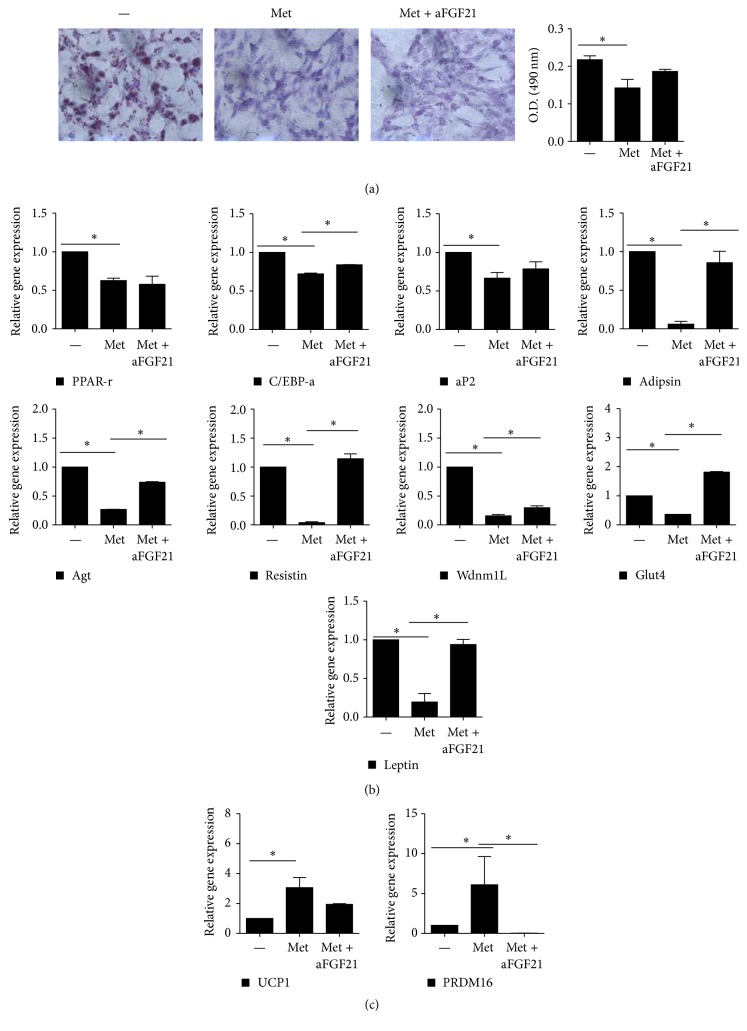
Metformin increased brown fat gene expression and function. (a) Oil Red O staining in metformin (5 mM) or metformin plus anti-FGF21-treated 3T3L1 cells. (b and c) Relative gene expression associated with (b) white adipose tissue (WAT) and (c) brown adipose tissue (BAT). Data represent means ± SEM.

To further confirm whether metformin affects FGF21 expression, we examined the size of lipid drops in metformin or metformin plus anti-FGF21-treated 3T3L1 adipocytes using Oil Red O staining. Lipid drop formation was unaffected by the use of anti-FGF21 (Figure 5(a)). We also observed the mRNA expression levels of WAT- and BAT-associated genes in the 3T3L1 adipocytes. The expression levels of the WAT-associated genes, such as C/EBPa, ap2, Adipsin, Agtl, Resistin, Glut4, and Leptin, significantly decreased in the metformin-treated group compared to the metformin plus anti-FGF21-treated group. In addition, the expression levels of BAT-associated genes, such as UCP1 and PRDM16, increased after metformin treatment (Figures 5(b) and 5(c)).

### 3.6. Effect of Metformin Treatment on Th17/Treg Balance in Mice with High-Fat Diet-Induced Obesity

**Figure 6:**
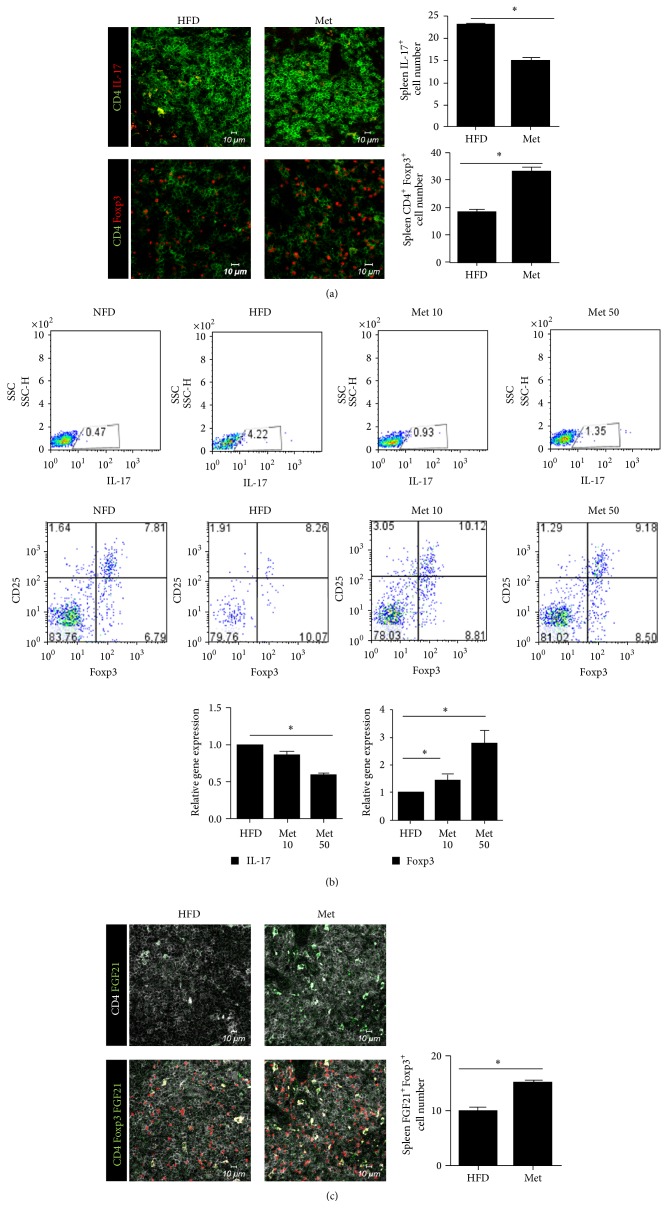
Effect ofmetformin on Th17/Treg cell regulation in obese mice. (a) CD4-positive IL-17-positive (Th17) cells and CD4-positive Foxp3-positive (Treg) cells shown by immunostaining spleen tissues in untreated obese mice and metformin-treated obese mice (original magnification, ×400). (b) The populations of Th17 and Treg cells were analyzed using flow cytometry and mRNA expression was determined using real-time PCR. (c) Spleen tissues from untreated obese mice and metformin-treated obese mice were observed by immunostaining for CD4-positive, Foxp3-positive, and FGF21-positive cells. Scale bars, 10 *μ*M (original magnification, ×400).

We next examined the effect of metformin treatment on helper T cell population. We counted the number of CD4-positive, IL-17-positive (Th17) cells, CD4-positive Foxp3-positive (Treg) cells, and CD4-positive Foxp3-positive FGF21-positive cells in the spleens of either obese or metformin-treated obese mice using confocal microscopy. Results showed that the number of Th17 cells decreased in metformin-treated obese mice. The number of Treg cells and FGF21-positive Treg cells was higher in the metformin-treated obese mice (Figures 6(a) and 6(c)). To confirm our results, the effect of metformin on Th17/Treg cell regulation in mice splenocytes was assessed using flow cytometry. The number of Th17 cells was lower in the metformin-treated obese mice than that in the untreated obese mice. The mRNA expression of the Th17-associated gene, IL-17, and the Treg-associated gene, Foxp3, was measured. IL-17 gene expression decreased and Foxp3 gene expression increased after metformin treatment (Figure 6(b)). Therefore, our results suggest that metformin has a beneficial effect on obesity.

## 4. Discussion

Metformin has been shown to be an effective antidiabetic drug and also shown to be useful against obesity. However, the mechanism of metformin and its effect on FGF21 in obesity is yet to be reported.

The most interesting finding of this research is that the antiobesity activity of metformin is related to its upregulation of FGF21 production. It is well documented that the levels of cholesterol, blood glucose, TG, and LDL are higher in obese patients than in healthy controls [16]. A significant downregulation of the levels of obesity-related factors including cholesterol, TG, and LDL was shown in the serum of metformin-treated obese mice. Since FGF21 has been shown to be a key regulator of obesity [11, 17], the enhancement of FGF21 expression has therapeutic potential. Therefore, our observations suggest that metformin has an antiobesity effect because it induces FGF21 production.

Metabolic markers including cholesterol, LDL, TG, and blood glucose are important factors in obesity. It is well known that high levels of TG and low levels of HDL are observed in visceral obesity [2]. Previous reports have suggested that the inhibition of cholesterol, LDL, TG, and blood glucose indicates an improvement in metabolic disorders related to obesity [18, 19]. Our findings revealed that cholesterol, LDL, TG, and blood glucose levels decreased after metformin treatment in a mouse model of obesity. Thus, metformin has the potential to ameliorate the metabolic dysfunction that is induced by obesity.

In obese individuals, fatty liver, white fat/brown fat balance, and fat mass are important factors. The metabolic variations linked to obesity cause fatty liver and increase fat mass in the body [3, 20]. In addition, the promotion of BAT and the inhibition of WAT are essential for the treatment of obesity [21]. In this study, metformin downregulated fatty liver and visceral fat. Moreover, metformin reduced the expression levels of WAT-associated genes such as PPAR-r, aP2, and Agt. Furthermore, the expression levels of BAT-associated genes including UCPI increased after metformin treatment. These results demonstrated that metformin has the potential to treat obesity.

Lipid droplets are cellular organelles that store neutral lipids within cells and play a key role in metabolic disease. It has been demonstrated that lipid droplets induce the pathogenesis involved in lipid aggregation such as obesity [22]. Since obesity is related to numerous inflammatory states, many inflammatory mediators are involved in obesity. For example, the number of Th17 cells increased in obesity adipose tissues and obesity exaggerates inflammation via Th17 differentiation [23]. It has been demonstrated that the level of several inflammatory cytokines increases owing to obesity [24–26]. Several investigations have shown that the TG levels in lipid droplets increase owing to obesity and lipid droplets induce an inflammatory response [27–29]. This study revealed that metformin treatment suppresses the number of lipid droplets in liver tissues and in HepG2 cells. These results indicate the anti-inflammatory effect of metformin.

The role of metformin in inflammatory response has already been investigated in various studies. It has been suggested that metformin ameliorates obesity and improves metabolic dysfunction by reducing body weight and fat mass [30–32]. It is also reported that metformin treatment induces FGF21 production [33, 34]. However, this study revealed that metformin attenuates inflammation through the induction of FGF21.

## 5. Conclusions

We have demonstrated that metformin suppresses high-fat diet-induced obesity and the associated inflammatory response by inducing FGF21 production in obese mice and HepG2 cells. This suggests that metformin could be a useful treatment of obesity.

## Abbreviations

FGF21: Fibroblast growth factor 21
AST: Aspartate transaminase
ALT: Alanine transaminase
TG: Triglyceride
CoxIV: Cytochrome c oxidase subunit 4
ITT: Insulin tolerance test
GTT: Glucose tolerance test
WAT: White adipose tissue
BAT: Brown adipose tissue
IL: Interleukin.

## References

[1] Després J.-P., Moorjani S., Tremblay A. (1989). Relation of high plasma triglyceride levels associated with obesity and regional adipose tissue distribution to plasma lipoprotein-lipid composition in premenopausal women. *Clinical and Investigative Medicine*.

[2] Tchernof A., Lamarche B., Prud'homme D. (1996). The dense LDL phenotype: association with plasma lipoprotein levels, visceral obesity, and hyperinsulinemia in men. *Diabetes Care*.

[3] Fabbrini E., Sullivan S., Klein S. (2010). Obesity and nonalcoholic fatty liver disease: biochemical, metabolic, and clinical implications. *Hepatology*.

[4] Patel C., Ghanim H., Ravishankar S. (2007). Prolonged reactive oxygen species generation and nuclear factor-kappaB activation after a high-fat, high-carbohydrate meal in the obese. *Journal of Clinical Endocrinology and Metabolism*.

[5] Manning P. J., Sutherland W. H. F., McGrath M. M., De Jong S. A., Walker R. J., Williams M. J. A. (2008). Postprandial cytokine concentrations and meal composition in obese and lean women. *Obesity*.

[6] Haslam D. W., James W. P. T. (2005). Obesity. *The Lancet*.

[7] Crowson C. S., Matteson E. L., Davis J. M., Gabriel S. E. (2013). Contribution of obesity to the rise in incidence of rheumatoid arthritis. *Arthritis Care and Research*.

[8] Winer S., Paltser G., Chan Y. (2009). Obesity predisposes to Th17 bias. *European Journal of Immunology*.

[9] Son H.-J., Lee J., Lee S.-Y. (2014). Metformin attenuates experimental autoimmune arthritis through reciprocal regulation of Th17/Treg balance and osteoclastogenesis. *Mediators of Inflammation*.

[10] Koh S.-J., Kim J. M., Kim I.-K., Ko S. H., Kim J. S. (2014). Anti-inflammatory mechanism of metformin and its effects in intestinal inflammation and colitis-associated colon cancer. *Journal of Gastroenterology and Hepatology*.

[11] Kharitonenkov A., Shiyanova T. L., Koester A. (2005). FGF-21 as a novel metabolic regulator. *The Journal of Clinical Investigation*.

[12] Coskun T., Bina H. A., Schneider M. A. (2008). Fibroblast growth factor 21 corrects obesity in mice. *Endocrinology*.

[13] Gälman C., Lundåsen T., Kharitonenkov A. (2008). The Circulating Metabolic Regulator FGF21 Is Induced by Prolonged Fasting and PPAR*α* Activation in Man. *Cell Metabolism*.

[14] Arner P., Pettersson A., Mitchell P. J., Dunbar J. D., Kharitonenkov A., Rydén M. (2008). FGF21 attenuates lipolysis in human adipocytes—a possible link to improved insulin sensitivity. *FEBS Letters*.

[15] Foltz I. N., Hu S., King C. (2012). Treating diabetes and obesity with an FGF21-mimetic antibody activating the *β*Klotho/FGFR1c receptor complex. *Science Translational Medicine*.

[16] Cowin I., Emmett P. (2000). Cholesterol and triglyceride concentrations, birthweight and central obesity in pre-school children. ALSPAC Study Team. Avon Longitudinal Study of Pregnancy and Childhood. *International Journal of Obesity and Related Metabolic Disorders*.

[17] Woo Y. C., Xu A., Wang Y., Lam K. S. L. (2013). Fibroblast growth factor 21 as an emerging metabolic regulator: clinical perspectives. *Clinical Endocrinology*.

[18] Fan S., Zhang Y., Sun Q. (2014). Extract of okra lowers blood glucose and serum lipids in high-fat diet-induced obese C57BL/6 mice. *Journal of Nutritional Biochemistry*.

[19] Dashti H. M., Al-Zaid N. S., Mathew T. C. (2006). Long term effects of ketogenic diet in obese subjects with high cholesterol level. *Molecular and Cellular Biochemistry*.

[20] Deurenberg P., Yap M. D., Wang J., Lin F. P., Schmidt G. (1999). The impact of body build on the relationship between body mass index and percent body fat. *International Journal of Obesity*.

[21] Seale P., Lazar M. A. (2009). Brown fat in humans: turning up the heat on obesity. *Diabetes*.

[22] Krahmer N., Farese R. V., Walther T. C. (2013). Balancing the fat: Lipid droplets and human disease. *EMBO Molecular Medicine*.

[23] Chen Y., Tian J., Tian X. (2014). Adipose tissue dendritic cells enhances inflammation by prompting the generation of Th17 cells. *PLoS ONE*.

[24] Hotamisligil G. S., Arner P., Caro J. F., Atkinson R. L., Spiegelman B. M. (1995). Increased adipose tissue expression of tumor necrosis factor-alpha in human obesity and insulin resistance. *The Journal of Clinical Investigation*.

[25] Shoelson S. E., Lee J., Goldfine A. B. (2006). Inflammation and insulin resistance. *Journal of Clinical Investigation*.

[26] Berg A. H., Scherer P. E. (2005). Adipose tissue, inflammation, and cardiovascular disease. *Circulation Research*.

[27] Bozza P. T., Viola J. P. B. (2010). Lipid droplets in inflammation and cancer. *Prostaglandins Leukotrienes and Essential Fatty Acids*.

[28] Beller M., Thiel K., Thul P. J., Jäckle H. (2010). Lipid droplets: a dynamic organelle moves into focus. *FEBS Letters*.

[29] McDonough P. M., Agustin R. M., Ingermanson R. S. (2009). Quantification of lipid droplets and associated proteins in cellular models of obesity via high-content/high-throughput microscopy and automated image analysis. *Assay and Drug Development Technologies*.

[30] Virtanen K. A., Hällsten K., Parkkola R. (2003). Differential effects of rosiglitazone and metformin on adipose tissue distribution and glucose uptake in type 2 diabetic subjects. *Diabetes*.

[31] Golay A. (2008). Metformin and body weight. *International Journal of Obesity*.

[32] Baptista T., Rangel N., Fernández V. (2007). Metformin as an adjunctive treatment to control body weight and metabolic dysfunction during olanzapine administration: a multicentric, double-blind, placebo-controlled trial. *Schizophrenia Research*.

[33] Nygaard E. B., Vienberg S. G., Ørskov C., Hansen H. S., Andersen B. (2012). Metformin stimulates FGF21 expression in primary hepatocytes. *Experimental Diabetes Research*.

[34] Kim K. H., Jeong Y. T., Kim S. H. (2013). Metformin-induced inhibition of the mitochondrial respiratory chain increases FGF21 expression via ATF4 activation. *Biochemical and Biophysical Research Communications*.

